# Properties of white matter tract diffusivity in children with developmental dyslexia and comorbid attention deficit/hyperactivity disorder

**DOI:** 10.1186/s11689-023-09495-9

**Published:** 2023-08-08

**Authors:** Ryan J. Slaby, C. Nikki Arrington, Jeffrey Malins, Rose A. Sevcik, Kenneth R. Pugh, Robin Morris

**Affiliations:** 1 Department of Psychology, Georgia State University, 140 Decatur St SE, Atlanta, GA 30303 USA; 2 GSU/Georgia Tech Center for Advanced Brain Imaging, 831 Marietta St NW, Atlanta, GA 30318 USA; 3 Department of Psychology, University of Milano-Bicocca, Piazza Dell’ Ateneo Nuovo,1, 20126 Milan, Italy; 4 Georgia State University, Center for Translational Research in Neuroimaging and Data Science, 55 Park Place, 18th Floor, Atlanta, GA 30303 USA; 5 Yale University, Haskins Laboratories, 300 George Street, Suite 900, New Haven, CT 06511 USA

**Keywords:** Dyslexia, ADHD, Comorbidity, DTI, White matter, SLF, FA, Reading, Attention, Executive function

## Abstract

**Background:**

Developmental dyslexia (DD) and attention deficit/hyperactivity disorder (ADHD) are highly comorbid neurodevelopmental disorders. Individuals with DD or ADHD have both been shown to have deficits in white matter tracts associated with reading and attentional control networks. However, white matter diffusivity in individuals comorbid with both DD and ADHD (DD + ADHD) has not been specifically explored.

**Methods:**

Participants were 3^rd^ and 4^th^ graders (age range = 7 to 11 years; SD = 0.69) from three diagnostic groups ((DD (*n* = 40), DD + ADHD (*n* = 22), and typical developing (TD) (*n* = 20)). Behavioral measures of reading and attention alongside measures of white matter diffusivity were collected for all participants.

**Results:**

DD + ADHD and TD groups differed in mean fractional anisotropy (FA) for the left and right Superior Longitudinal Fasciculus (SLF)-Parietal Terminations and SLF-Temporal Terminations. Mean FA for the DD group across these SLF tracts fell between the lower DD + ADHD and higher TD averages. No differences in mean diffusivity nor significant brain-behavior relations were found.

**Conclusions:**

Findings suggest that WM diffusivity in the SLF increases along a continuum across DD + ADHD, DD, and TD.

## Background

Although reading occurs relatively easily for most children, the 5–17% of children who cannot learn to read proficiently may be affected by developmental dyslexia (DD; [1–3]). DD is a neurodevelopmental disorder that is defined by difficulty in processing phonological information [2, 4] along with deficits in rapid automatic naming [5]. DD is also associated with poor reading fluency and comprehension in comparison to typically developing peers. Many of these deficits found within DD are linked to impairments in the well-defined left-hemisphere language and reading network [1, 6]. Other deficits commonly related to DD include poor short-term and working memory [7], difficulties with visual information processing [8], and slowed processing speed [9], all of which are seen in children with other neurodevelopmental disorders.

Research indicates that approximately 40% of children with DD also meet criteria for at least one additional disability [10, 11], with attention deficit/hyperactivity disorder (ADHD) being the most commonly co-occurring impairment, diagnosed in 25–40% of children with DD [11]. A majority of these comorbid ADHD cases fall within the inattentive subtype of ADHD (ADHD-I; 11). The prevalence of comorbid inattentive behaviors is significantly higher than would be expected by chance, with up to 26% of individuals with DD also meeting criteria for ADHD-I [11, 12]. Moreover, genetic overlaps have been reported between DD and ADHD-I [13–16]. Inattention in these children has also been shown to negatively impact behavior and academic performance [17, 18], and those students with DD plus co-morbid ADHD-I may suffer from deficits in attention that additionally impede their reading development [17, 19]. Children experiencing greater inattention typically perform poorly on math and reading achievement tests, even after controlling for intelligence [12, 20–22]. Furthermore, a strong relationship between attention and the development of pre-reading skills in preschoolers may later impact the development of word identification abilities [23]. The combination of DD and ADHD disorders therefore may impact a child’s reading development above that of either DD or ADHD when diagnosed exclusively [9, 24–26].

### Neural correlates

Although much research has characterized DD and ADHD as two separate and distinct disorders [27, 28], DD and ADHD may share components across their underlying neural systems, which may account for their higher rate of comorbidity. Research has identified a complex neural reading network, consisting of a predominantly left–hemisphere system that encompasses the inferior frontal, temporoparietal, and occipitotemporal cortical regions [29, 30]. Three distinct neural pathways, or subsystems, have been shown to work in parallel to accomplish fluent and proficient reading [2, 31]. The reading network’s dorsal system is comprised of left temporoparietal areas including the angular gyrus, supramarginal gyrus, and posterior portions of the superior temporal gyrus, which are thought to play a role in mapping orthographic information to the phonological and semantic properties of written words [32]. The ventral system is associated with the left ventral occipitotemporal cortex extending into the middle and inferior temporal gyrus, which facilitates processing of the orthographic features of written language that is necessary for automatic word recognition [33, 34]. The anterior system is focused within the left inferior frontal gyrus (IFG) and adjacent frontal gyri and is important for several processes such as phonological recoding and semantic integration [35–37]. Individuals with DD show reduced functional [38] and network connectivity [39] within the ventral subsystem. Moreover, network connectivity within the ventral system improves with reading skills over time, yet dorsal system network connectivity decreases over time with improved reading skills [39].This suggests an increased reliance on the automatic ventral subsystem and reduced reliance on the phonological dorsal subsystem with improved reading.

Research has deemed adequate attentional control as necessary for efficient executive functioning [40, 41], and children with DD show impairments in selective attention and the executive functions of inhibition and working memory [42]. In the same vein, neuroimaging research connected to attention and ADHD has identified a cingulo–fronto–parietal attentional control network, which is further associated with the fronto–striatal and fronto–parietal pathways [43]. Indeed, this attentional control network consists of connections between the lateral frontal pole, anterior cingulate cortex, dorsolateral prefrontal cortex, ventrolateral prefrontal cortex, inferior parietal lobe, and various subcortical regions [44]. As a primary substrate for attention and executive functioning [43], this network is thought to facilitate goal–directed processes and provides for the ability to respond to changing task demands [44]. Individuals with attentional deficits, such as those associated with ADHD, show decreased activation within the attentional control network [45, 46]. Specifically, individuals with deficits in attentional control have shown hypoactivation in brain areas associated with both attention and executive function such as the anterior cingulate cortex, the parietal cortex, the ventrolateral prefrontal cortex, and the dorsolateral prefrontal cortex.

These distinct, yet overlapping, neural systems attributed to DD and ADHD may at times share a range of neural deficits between the two disorders, leading to the commonly occurring co-morbid DD and ADHD (DD + ADHD). Indeed, individuals with both a reading disability (encompassing DD) and ADHD have shown gray matter differences within regions of the frontal-striatal pathway partly comprising the attentional control network [47] and the reading network [48], which have been functionally associated with executive functioning and reading ability, respectively [49]. Likewise, a meta-analysis found similar regional deficits within the frontal-striatal pathways in DD and ADHD populations [50]. Moreover, research shows overlapping gray matter correlates within the attentional control network for ADHD and comorbid reading disability and ADHD [47] and within the reading network for reading disability and comorbid reading disability and ADHD [48, 49]. Therefore, it may be expected that those children who show co-morbid DD + ADHD attributes would show increasing levels of deficits among those shared components of the neural systems.

### White matter tracts of interest

These complex reading and attentional networks require effective and rapid communication between their connected regions to function effectively. Therefore, at a basic structural level, it is important to understand the quality and role of these network’s connecting white matter tracts and their relationship with specific behavioral outcomes in children with DD. Diffusion tensor imaging** (**DTI), which allows for the quantification of diffusion properties for white matter [51], permits such an examination of the structural pathways that underlie the reading and attentional networks. Quantitative analysis yields many different diffusivity-based measures of white matter with both fractional anisotropy (FA) and mean diffusivity (MD) being widely used [52]. FA utilizes eigenvalues that quantify diffusivity parallel and perpendicular to tract fibers to measure the fraction of the “magnitude” of anisotropic diffusion, which quantifies the degree of directionality for diffusivity [52, 53]. MD is the average of principal diffusivities parallel and perpendicular to the axon [54] with higher MD reflecting higher diffusivity along the axon. As markers of white matter diffusivity, FA and MD are useful quantities to compare across subjects as they provide information about directional architecture and axonal myelination [55].

**Fig. 1:**
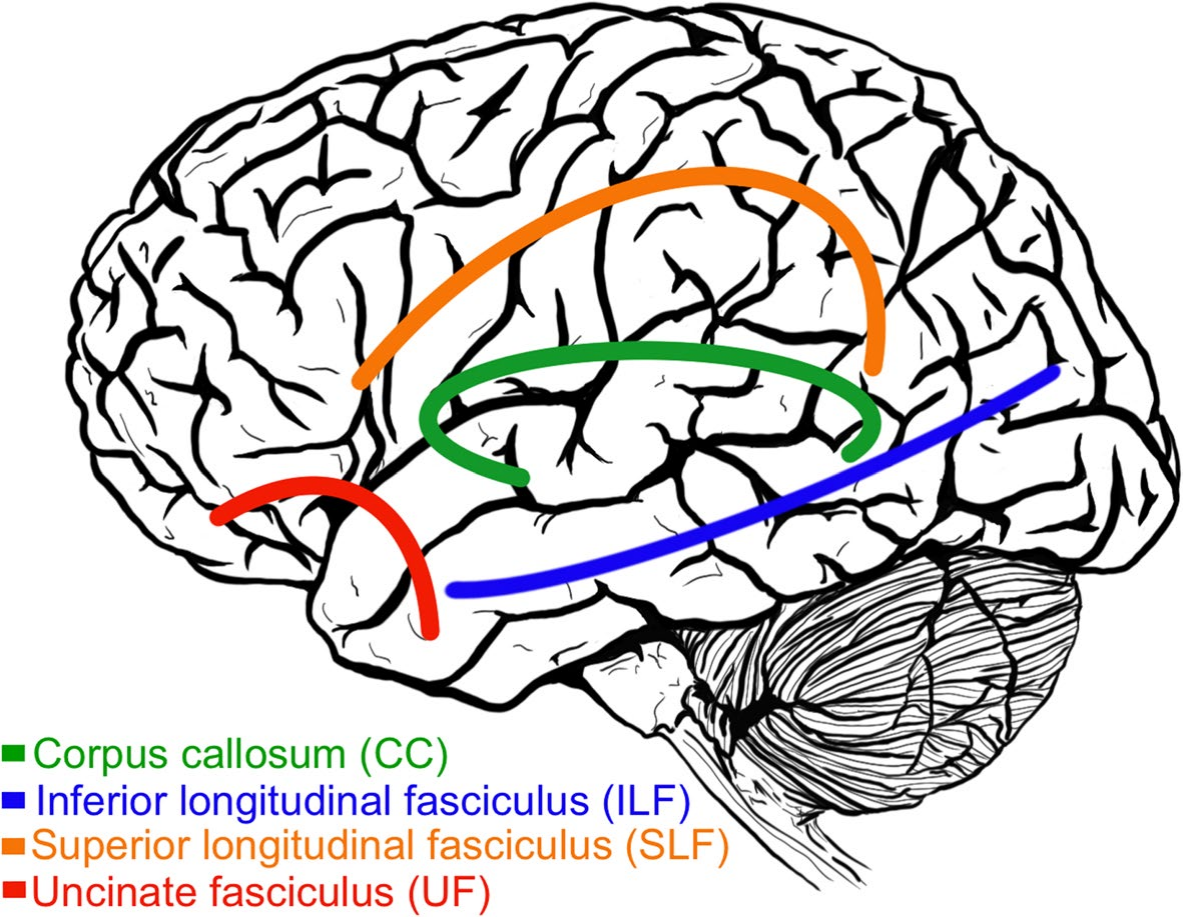
White matter tracts of interest Note: green = corpus callosum (CC); blue = inferior longitudinal fasciculus (ILF); orange = superior longitudinal fasciculus (SLF); red = uncinate fasciculus (UF)

Four primary white matter tracts (Fig. 1) – namely, the superior longitudinal fasciculus (SLF), the inferior longitudinal fasciculus (ILF), the uncinate fasciculus (UF), and regions of the corpus callosum (CC) – have been closely studied within both the reading [2] and attentional control [43] networks. The SLF is a large lateral associative white matter bundle generally connecting the temporoparietal area (TPA) with the frontal, parietal and temporal areas and may segmented into three branches: (1) the dorsal SLF, a direct segment running medially, connecting the TPA with the middle (MFG) and superior frontal gyri; (2) the ventral SLF, the lateral anterior segment linking the IFG and MFG to the inferior parietal lobule (IPL); (3) the posterior SLF, linking the TPA with the IPL via the lateral indirect posterior segment [56]. The ventral and dorsal SLF, subsuming portions of the arcuate fasciculus (AF), are typically thought to constitute the SLF temporal bundle (SLFt), whereas the posterior SLF constitutes the SLF parietal bundle (SLFp; [57]. Children and adults with poor word reading ability have shown decreased white matter diffusivity in the left SLFt [6] and SLFp [58] as indicated by lower FA values. Moreover, Chinese children with DD have been shown to have lower FA values in the left SLFt, associated with phonological processing skills [59]. Individuals with ADHD have also been shown to have reduced FA in the right SLF [60] and increased MD in the left SLF [61] compared to non-impaired controls.

The ILF is a ventral associative tract consisting of long and short fibers that directly connect the occipital and anterior temporal lobes [62]. Research has suggested relations between the left ILF and measures of word reading fluency [63, 64] and reading comprehension [63, 65]. Decreased FA in the left ILF has been reported in Chinese children with DD and this was related to semantic skills as appropriate for the Chinese logographic alphabet [66]. Reduced diffusivity in the ILF, bilaterally, has also been shown in children with ADHD [67]. Moreover, reduced FA in the left ILF has been observed in adults with ADHD, whereas MD in the left ILF has shown a negative association with attentional performance [68].

The UF is a ventral association bundle that connects the anterior temporal lobe with the orbitofrontal cortex, including the IFG [69]. It is thought to play a role in language functions such as lexical retrieval and semantic associations [70] with research implicating a role in reading comprehension [65, 71]. DD has been associated with reduced white matter connectivity in the UF [72]. Conversely, increased FA [61, 73] and MD [61] values in the bilateral UF have been observed in adults with the combined inattentive and hyperactive ADHD subtype (ADHD-C).

The CC is a major commissure that connects the left and right hemispheres of the brain and is mainly associated with interhemispheric connectivity [74]. Based on a multivariate machine learning approach, the CC is one of the most discriminative features classifying DD [75], and research has shown that children’s reading skill is negatively correlated with FA within the posterior callosum across typical and impaired readers [76]. For individuals with ADHD, measures of inattention and hyperactivity have been negatively correlated with a reduced cortical thickness of the CC in older adults [77] and lower FA values in the CC for children [78]. Most notably, a meta-analysis of white matter diffusivity in children and adults with ADHD found lower FA in the right forceps minor of the CC in comparison to those without ADHD [79].

### Current study

Researchers have investigated the relations between white matter tract diffusivity of the CC, ILF, SLF, and UF as they relate to groups of individuals diagnosed with either DD or ADHD independently but have not evaluated their role in comorbid subjects who have DD + ADHD. The primary aim of this study was to explore potential differences in white matter tract diffusivity of the CC, ILF, SLF, and UF in children with DD only, DD + ADHD, and compared to typically developing, unimpaired readers (TD). We also sought to investigate the relations between measures of white matter diffusivity and behavioral measures of reading ability and attentional control in these groups. We hypothesized that white matter diffusivity for the tracts of interest would be significantly reduced in DD + ADHD compared to both DD and TD groups due to their shared underlying neural deficits, and based on the previous literature, that the DD only group would show reduced white matter diffusivity compared to the TD group. Furthermore, we hypothesized that white matter tract diffusivity would be positively correlated with measures of reading proficiency and attentional control.

## Methods

### Participants

Participants were recruited from public and charter elementary schools in the greater Atlanta area as part of a longitudinal study of reading intervention approved by the Georgia State University/Georgia Tech Center for Advanced Brain Imaging Institutional Review Board. All parents/students provided informed consent/assent before any participation in the study. Participants included 3rd and 4th grade students from 7 to 11 years old (mean age = 9.32; SD = 0.69; please see Table 1 for age statistics separated by group) who completed baseline behavioral/cognitive assessments and an MRI scan (including DWI sequences) as part of participation in the larger study. Participants were assigned to one of three groups based on their reading disability and ADHD comorbidity status: DD, DD + ADHD, or TD. Children identified with DD (*n* = 40) scored at least one standard deviation below age-norm expectations on any of the following: *Woodcock Johnson 3*^*rd*^* Edition* (WJ-3; [80]) Broad Reading Cluster subtests or the composite, the WJ-3 Basic Reading Cluster subtests or composite, or *Test of Word Reading Efficiency 2*^*nd*^* Edition* (TOWRE-2; [81]) subtests. DD + ADHD readers (*n* = 22) met the same criteria for DD and also exhibited high ADHD symptomology as defined by the *Strengths and Weaknesses of ADHD symptoms and Normal Behavior* (SWAN; [82]) and the *Disruptive Behavior Rating Scale* (DBRS; [83]) as rated by a guardian and teacher. Guardians and classroom teachers were asked to complete both the SWAN and DBRS rating scales on all participants in the study. A composite of these scores and individual symptom ratings on these scales were used to identify subjects in the DD + ADHD group using current DSM-5 criteria for both the Combined and Inattentive types. In the rare case that data was not returned from one rater (i.e., parent or teacher) or rating scale, available scores were used. TD readers (*n* = 20) were recruited from the same schools but did not meet criteria for either DD or ADHD. All participants had a verbal and/or performance intelligence standard score at or above 80 on one of the subtests of the *Wechsler Abbreviated Scale of Intelligence—II* (WASI-II; [84]) in order to rule out intellectual disabilities. All children in the study completed screening materials for diagnostic criteria and were native English speakers. Children with chronic absenteeism (> 15 absences per year), hearing impairment (< 20/40), serious emotional/psychiatric disturbance, chronic medical/neurological condition, or MRI contraindicative according to guardian report were excluded.

### Behavioral measures

#### Reading measures

The composite score of the Sight Word Efficiency and Phonological Decoding Efficiency subtests of the TOWRE-2 was used as a measure of word reading fluency (TOWRE-CST). The TOWRE-2 requires participants to read aloud words and pseudowords, respectively, as quickly and as accurately as possible. A higher TOWRE-CST reflects better word reading efficiency.

#### Attentional and Executive Function measures

The Behavior Rating Inventory of Executive Function (BRIEF) measured executive function via an 86-item questionnaire answered by a parent or guardian [85]. The Global Executive Composite (GEC) T Score is a combined measure of all sub-scales produced by BRIEF and provides a measure of executive function (GEC-T). A higher GEC-T reflects poorer executive function. The Behavioral Regulation Index (BRI) T Score is a combined measure of the Inhibit, Shift, and Emotional Control subscales and provides a measure of behavioral attention (BRI-T). A higher BRI-T reflects poorer behavioral attention.

### Magnetic resonance imaging

#### Data acquisition MRI

Images were acquired using a 3 T Siemens scanner located at the GSU/GaTech Center for Advanced Brain Imaging in Atlanta, Georgia. The site scanner was upgraded from a Trio (12-channel head coil) to a PRIMSA-Fit (20-channel) during the final year of data collection (*n* = 13). Data acquisition and scan parameters were kept consistent throughout the duration of the study and processed data was harmonized to account for inter-scanner differences (see Imaging data preprocessing section below). All included subjects completed diffusion-weighted imaging data, collected in two separate sequences with reverse phase encoding (anterior-to-posterior and posterior-to-anterior) via the following parameters: FoV: 220 × 220 mm; slice thickness: 2 mm; repetition time TD/TE: 8900/97 ms; slices: 64; b:1000, 4* b:0; 32 gradient directions; voxel size isotropic: 2 mm. Total DWI acquisition time for collection of both sequences was approximately 10 min total. T2*-weighted images were acquired in an axial-oblique orientation parallel to the intercommissural line (32 slices; 4 mm slice thickness; no gap) using single-shot echo planar imaging (matrix size = 64 × 64; voxel size = 3.438 × 3.438 × 4 mm; FoV = 220 mm; TD = 2000 ms; TE = 30 ms; flip angle = 80°). Anatomical scans were collected in the same orientation (MPRAGE; matrix size = 256 × 256; voxel size = 1 × 1 × 1 mm; FoV = 256 mm; TD = 2530 ms; TE = 2.77 ms; flip angle = 7°).

#### Imaging data preprocessing

After visual and automated quality assurance for all image data, DWI data were preprocessed with TORTOISE [86] using a T2* structural file and a MPRAGE reorientation file. DIFF_PREP was used for motion and eddy current distortion with computed B-matrix of gradient tables [87]. DR-BUDDI corrected susceptibility induced EPI distortions via blip-up and blip-down data (AP/PA co-registration), and DWIs were reoriented into target space with B-matrices [88].

The FreeSurfer 6.0 image analysis suite was used to process anatomical data [89]. This automated procedure contained segmentation of cortical and subcortical white matter, tessellation of gray matter/white matter boundaries, inflation of the folded surface tessellation patterns [90, 91], and automatic correction of topographical defects [92]. Manual intervention was performed by a trained technician consistent with FreeSurfer protocol, when necessary.

Automated reconstruction of white matter tracts of interest was carried out via FreeSurfer’s TRACULA pipeline using global probabilistic tractography [57]. Specifically, the combination of FreeSurfer’s cortical parcellation and subcortical segmentation with TRACULA’s anatomical atlas provided the automated reconstruction of 18 major white matter tracts. TRACULA utilizes FSL’s bedpostx to fit the ball-and-stick model to DWI data and reconstruct pathways. Out of the 18 tracts, we extracted the FA and MD values for the following white matter tracts of interest (L = left; R = right): the SLFt (SLF temporal bundle, consisting of components of ventral and dorsal SLF and AF), the SLFp (SLF parietal bundle; SLF-posterior), the ILF, the UF, the fminor (anterior CC; forceps minor), and fmajor (posterior CC; forceps major).

To account for differences between data collected before and after scanner upgrade, DTI data were harmonized using ComBat [93]. ComBat assumes the imaging feature measurements can be modeled as a linear combination of the biological variables with the scanner effects as an error term that includes a multiplicative scanner-specific scaling factor. It has been shown to effectively reduce inter-scanner variation in DTI data while effectively preserving biological associations [94, 95].

### Statistical analyses

A separate one-way between subjects analysis of variance compared the TOWRE-CST, BRI-T, and GEC-T on three levels: DD + ADHD, DD, and TD. Likewise, separate one-way between subject analysis of variance models compared the harmonized means for FA and MD for each of the tracts of interest individually on three levels: DD, DD + ADHD, and TD. Mean FA and MD data were assessed for extreme outliers via box-and-whisker plots, which resulted in the removal of two outliers that were present in more than one white tract of interest. Participants section details our total sample size (*n* = 82) after outlier removal and for all data analyses. The Tukey–Kramer post hoc test was used to test for significant group differences for all analyses. Pearson bivariate correlations were run for mean FA and MD of the tracts of interest and the following behavioral measures: TOWRE-CST, BRI-T, and GEC-T. The False Discovery Rate (FDR) was applied to all correlations to correct for multiple comparisons.

## Results

### Behavioral comparisons between groups

The one-way analysis of variance revealed significant differences between groups on all behavioral measures of reading, attention, and intelligence (Tables 1 and 2): WJ-3 Basic (F(2,79) = 88.9, *p* < 0.001), WASI-II (F(2,79) = 31.0, *p* < 0.001), TOWRE-CST (F(2,79) = 93.5, *p* < 0.001), BRI-T (F(2,79) = 7.42, *p* < 0.001), and GEC-T (F(2,79) = 23.3, *p* < 0.001). For diagnostic measures, TD significantly differed from DD and DD + ADHD on the WJ-3- Basic (*p* < 0.001) and WASI-II (*p* < 0.001) showing the highest reading and intelligence scores, respectively; however, DD and DD + ADHD showed no significant differences. Likewise, TD significantly differed from DD and DD + ADHD on the TOWRE-CST (*p* < 0.001) showing the highest reading score; however, DD and DD + ADHD showed no significant differences. Moreover, DD + ADHD significantly differed from both TD (*p* < 0.001) and DD (*p* = 0.026) on the BRI-T showing the lowest behavioral attentional score; however, TD and DD showed no significant difference. On the GEC-T, TD significantly differed from DD (*p* = 0.014) and DD + ADHD (*p* < 0.001), while DD significantly differed from DD + ADHD ( *p* < 0.001) with DD + ADHD showing the lowest and TD showing the highest executive functioning score.

**Table 1:** ANOVA of individual group descriptive statistics with significant groups confirmed via Tukey–Kramer post-hoc tests

Variable	DD + ADHD *M (SD)*	DD *M (SD)*	TD *M (SD)*	*F*(2,79)	*p*	*η*_*p*_^*2*^	*Tukey–Kramer post-hoc tests*
*N* (Female)	22 (8)	40 (27)	20 (9)	--	--	--	--
Age	9.27 (0.68)	9.34 (0.76)	9.34 (0.56)	--	--	--	--
BRI-T	57.05 (10.86)	49.00 (13.16)	43.65 (7.54)	**7.42**	**<0 .001**	**0.158**	**TD < DD & DD + ADHD**
GEC-T	62.73 (7.69)	49.98 (12.36)	42.10 (5.96)	**23.3**	**< 0.001**	**0.371**	**TD < DD < DD + ADHD**
TOWRE-CST	69.32 (10.18)	72.85 (8.46)	105.45 (11.30)	**93.5**	**< 0.001**	**0.703**	**TD > DD & DD + ADHD**
WASI-II	92.96 (9.42)	91.93 (8.31)	112.25 (12.85)	**31.0**	**< 0.001**	**0.440**	**TD > DD & DD + ADHD**
WJ3-Basic	84.1 (8.83)	86.45 (8.17)	111.85 (6.32)	**82.9**	**< 0.001**	**0.692**	**TD > DD & DD + ADHD**

*M* Mean, *SD* Standard deviation, *Bolded* Statistically significant values, *DD* Developmental dyslexia, *DD* + *ADHD* Developmental dyslexia comorbid with attention deficit/hyperactivity disorder, *TD* Typical developing, unimpaired readers, *BRI-T* BEHAVIORAL Regulation Index T Score taken from the Behavior Rating Inventory of Executive Function (BRIEF), *GEC-T* Global Executive Composite T Score taken from BRIEF, *TOWRE-CST* Test of Word Reading Efficiency 2^nd^ Edition Composite Score, *WASI-II* Wechsler Abbreviated Scale of Intelligence – II, *WJ3-Basic* Woodcock Johnson-III Basic Reading Composite Score. Note: BRI-T differs for TD < DD + ADHD & DD < DD + ADHD; no difference between DD & TD. TOWRE-CST, WASI-II, & WJ3-Basic differ for TD > DD + ADHD & TD > DD + ADHD; no difference between DD & DD + ADHD

**Table 2:** Tukey–Kramer post-hoc test results from ANOVA of individual statistics for behavioral measures

Measure	Group Comparisons	Mean Difference	*p*	95% Confidence Interval	
				**Lower Bound**	**Upper Bound**
BRI-T	TD < DD	5.35	0.208	-2.12	12.82
	**TD < DD + ADHD**	**13.40**	**< 0.001**	**4.97**	**21.83**
	**DD < DD + ADHD**	**8.05**	**0.026**	**0.81**	**15.29**
GEC-T	**TD < DD**	**7.88**	**0.014**	**1.35**	**14.41**
	**TD < DD + ADHD**	**20.63**	**< 0.001**	**13.26**	**28.00**
	**DD < DD + ADHD**	**12.75**	**< 0.001**	**6.42**	**19.08**
TOWRE-CST	**TD > DD**	**32.60**	**< 0.001**	**26.27**	**38.93**
	**TD > DD + ADHD**	**36.13**	**< 0.001**	**28.99**	**43.27**
	DD > DD + ADHD	3.53	0.359	-2.60	9.66
WASI-II	**TD > DD**	**20.32**	**< 0.001**	**13.86**	**26.78**
	**TD > DD + ADHD**	**19.29**	**< 0.001**	**12.01**	**26.57**
	DD > DD + ADHD	-1.03	0.918	-7.29	5.23
WJ3-Basic	**TD > DD**	**25.40**	**< 0.001**	**20.20**	**30.60**
	**TD > DD + ADHD**	**27.75**	**< 0.001**	**21.88**	**33.62**
	DD > DD + ADHD	2.35	0.509	-2.69	7.39

*Bolded* Statistically significant values, *DD* Developmental dyslexia, *DD* + *ADHD* Developmental dyslexia comorbid with attention deficit/hyperactivity disorder, *TD* Typical developing, unimpaired readers, *BRI-T* Behavioral Regulation Index T Score taken from the Behavior Rating Inventory of Executive Function (BRIEF), *GEC-T* Global Executive Composite T Score taken from BRIEF, *TOWRE-CST* Test of Word Reading Efficiency 2^nd^ Edition Composite Score, *WASI-II* Wechsler Abbreviated Scale of Intelligence – II, *WJ3-Basic* Woodcock Johnson-III Basic Reading Composite Score

### White matter comparisons between groups

**Fig. 2:**
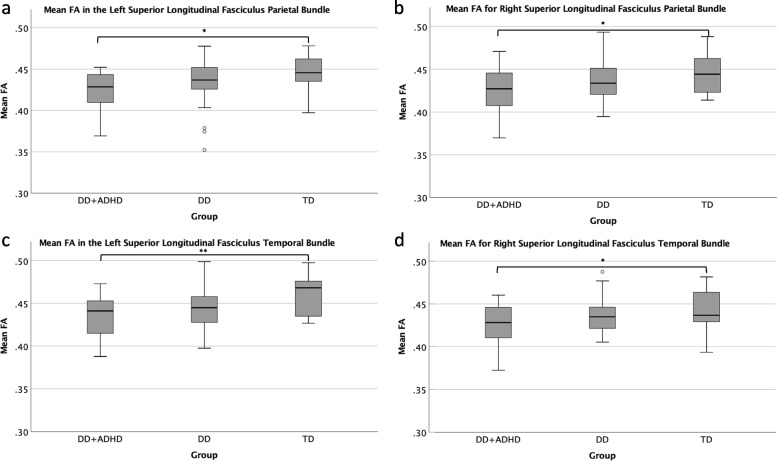
Box and whisker plots for tracts of interest showing significant differences in mean FA values between groups Note: * = *p* < .05; ** = *p* < .01; DD = developmental dyslexia; DD + ADHD = developmental dyslexia comorbid with attention deficit/hyperactivity disorder; TD = typical developing, unimpaired readers; FA = Fractional Anisotropy

The one-way analysis of variance revealed significant differences between groups for the mean FA values in the following tracts of interest (Tables 3 and 4): L SLFp (F(2,79) = 3.90, *p* = 0.024), L SLFt (F(2,79) = 3.13, *p* = 0.003), R SLFp (F(2,79) = 3.33, *p* = 0.041), and R SLFt (F(2,79) = 3.72, *p* = 0.029). There were significant differences in the mean FA of the L SLFp (*p* = 0.018), L SLFt (*p* = 0.002), R SLFp (*p* = 0.036), R SLFt (*p* = 0.029), such that the DD + ADHD group had significantly lower FA than the TD group in all tracts (Fig. 2). There were no significant differences for DD compared to DD + ADHD or between DD compared to TD. There were no significant differences between groups for mean MD for all tracts of interest.

**Table 3:** ANOVA of mean FA values for tracts of interest with significant groups confirmed via Tukey–Kramer post-hoc tests

Tract	DD + ADHD *(n* = 22*) M (SD)*	DD *(n* = 40*) M (SD)*	TD *(n* = 20*) M (SD)*	*F*(2,79)	*p*	*η*_*p*_^*2*^	*Tukey–Kramer post-hoc tests*
Fmajor	0.56 (0.05)	0.59 (0.05)	0.59 (0.04)	2.39	0.098	0.06	n/a
Fminor	0.52 (0.04)	0.53 (0.03)	0.51 (0.03)	1.26	0.290	0.03	n/a
L ILF	0.47 (0.03)	0.48 (0.03)	0.48 (0.03)	1.47	0.236	0.04	n/a
**L SLFp**	0.43 (0.02)	0.44 (0.03)	0.45 (0.02)	**3.90**	**0.024**	**0.08**	TD > DD + ADHD
**L SLFt**	0.43 (0.03)	0.45 (0.02)	0.46 (0.02)	**6.13**	**0.003**	**0.13**	TD > DD + ADHD
L UF	0.36 (0.03)	0.35 (0.06)	0.35 (0.06)	0.29	0.751	> 0.01	n/a
R ILF	0.47 (0.02)	0.48 (0.03)	0.48 (0.03)	1.51	0.228	0.04	n/a
**R SLFp**	0.43 (0.03)	0.44 (0.02)	0.45 (0.02)	**3.33**	**0.041**	**0.08**	TD > DD + ADHD
**R SLFt**	0.43 (0.02)	0.44 (0.02)	0.44 (0.02)	**3.72**	**0.029**	**0.09**	TD > DD + ADHD
R UF	0.37 (0.03)	0.38 (0.04)	0.37 (0.05)	0.13	0.879	> 0.01	n/a

*M* Mean, *SD* Standard deviation, Bolded Statistically significant values, *DD* Developmental dyslexia, *DD* + *ADHD* Developmental dyslexia comorbid with attention deficit/hyperactivity disorder, *TD* Typical developing, unimpaired readers, *FA* Fractional Anisotropy, *L* Left, *R* Right, *Fmajor* Forceps major, *Fminor* Forceps minor, *ILF* Inferior longitudinal fasciculus, *SLFp* Superior longitudinal fasciculus parietal bundle, *SLFt* Superior longitudinal fasciculus temporal bundle, *UF* Uncinate fasciculus

**Table 4:** Tukey–Kramer post-hoc test results from analysis of variance of mean FA values for tracts of interest

Tract	Group Comparisons	Mean Difference	*p*	95% Confidence Interval	
				**Lower Bound**	**Upper Bound**
L SLFp	TD > DD	0.010	0.269	-0.006	0.026
	**TD > DD + ADHD**	**0.021**	**0.018**	**0.003**	**0.039**
	DD > DD + ADHD	0.011	0.236	-0.005	0.026
L SLFt	TD > DD	0.015	0.076	-0.001	0.031
	**TD > DD + ADHD**	**0.026**	**0.002**	**0.008**	**0.044**
	DD > DD + ADHD	0.012	0.179	-0.004	0.027
R SLFp	TD > DD	0.007	0.551	-0.009	0.023
	**TD > DD + ADHD**	**0.019**	**0.036**	**0.001**	**0.037**
	DD > DD + ADHD	0.012	0.159	-0.003	0.027
R SLFt	TD > DD	0.005	0.637	-0.009	0.020
	**TD > DD + ADHD**	**0.018**	**0.029**	**0.001**	**0.034**
	DD > DD + ADHD	0.012	0.096	-0.002	0.026

*Bolded* Statistically significant values, *DD* Developmental dyslexia, *DD* + *ADHD* Developmental dyslexia comorbid with attention deficit/hyperactivity disorder, *TD* Typical developing, unimpaired readers, *FA* Fractional Anisotropy, *L* Left, *R* Right, *SLFp* Superior longitudinal fasciculus parietal bundle, *SLFt* Superior longitudinal fasciculus temporal bundle

### Brain—behavior relations

**Fig. 3:**
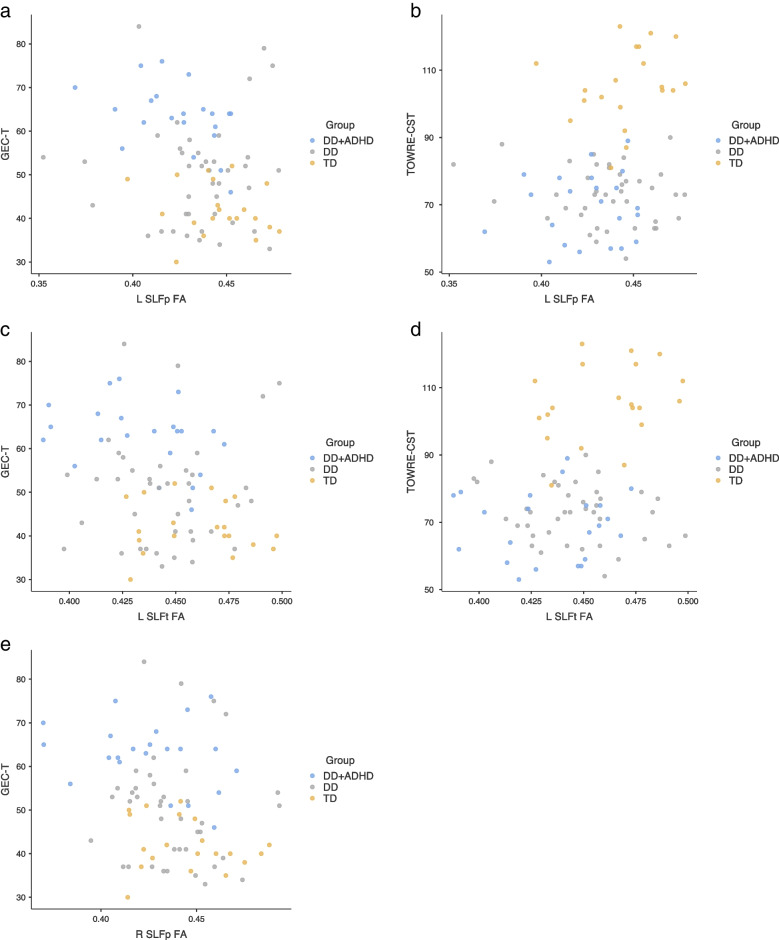
Scatterplots for brain behavior relations of significance before correcting for multiple comparisons within tracts of interest showing significant differences in mean FA values between groups Note: DD = developmental dyslexia; DD + ADHD = developmental dyslexia comorbid with attention deficit/hyperactivity disorder; TD = typical developing, unimpaired readers; GEC-T = Global Executive Composite T Score taken from the Behavior Rating Inventory of Executive Function; TOWRE-CST = Test of Word Reading Efficiency 2^nd^ Edition Composite Score; FA = Fractional Anisotropy; L = Left; R = Right; SLFp = superior longitudinal fasciculus parietal bundle; SLFt = superior longitudinal fasciculus temporal bundle

Pearson bivariate correlations revealed relations between behavioral measures and measures of white matter diffusivity for tracts of interest. Non-corrected significant correlations with mean FA included the following: TOWRE-CST and L SLFp (r (80) = 0.220, *p* = 0.047) and L SLFt (r (80) = 0.269, *p* = 0.015); BRI-T and L UF (r (80) = 0.229, *p* = 0.036); GEC-T and L SLFp (r (80) = -0.240, *p* = 0.030), L SLFt (r (80) = -0.232, *p* = 0.036), and R SLFp (r (80) = -0.263 *p* = 0.017). For mean MD, correlations were found between the GEC-T and L UF (r (80) = -0.233, *p* = 0.035); BRI-T and L UF (r (80) = -0.234, *p* = 0.034). However, no significant correlations were maintained after correction for multiple comparisons (Table 5) (Fig. 3).

**Table 5:** Pearson’s correlational matrix of brain-behavior relations before correcting for multiple comparisons

	**BRI-T**	**GEC-T**	**TOWRE-CST**
Fmajor FA	-0.08	-0.20	0.10
Fminor FA	-0.06	-0.04	-0.13
L ILF FA	-0.09	-0.18	0.09
L SLFp FA	-0.11	**-0.24**	**0.22**
L SLFt FA	-0.12	**-0.23**	**0.27**
L UF FA	**0.23**	0.21	-0.04
R ILF FA	-0.04	-0.13	0.06
R SLFp FA	-0.17	**-0.26**	0.16
R SLFt FA	-0.03	-0.15	0.18
R UF FA	0.15	0.14	-0.08
Fmajor MD	-0.02	0.09	0.09
Fminor MD	-0.15	-0.12	0.19
L ILF MD	-0.07	0.04	0.18
L SLFp MD	-0.06	0.02	0.06
L SLFt MD	-0.09	0.01	0.11
L UF MD	**-0.23**	**-0.23**	0.13
R ILF MD	-0.04	0.06	0.12
R SLFp MD	-0.07	> 0.01	0.02
R SLFt MD	-0.09	0.02	0.05
R UF MD	-0.21	-0.14	0.10

*Bolded* statistically significant values (*p* < 0.05) before correcting for multiple comparisons, *BRI-T* Behavioral Regulation Index T Score taken from the Behavior Rating Inventory of Executive Function (BRIEF), *GEC-T* Global Executive Composite T Score taken from BRIEF, *TOWRE-CST* Test of Word Reading Efficiency 2^nd^ Edition Composite Score, *FA* Fractional Anisotropy, *MD* Mean Diffusivity, *L* Left, *R* Right, *Fmajor* Forceps major, *Fminor* Forceps minor, *ILF* inferior longitudinal fasciculus, *SLFp* Superior longitudinal fasciculus parietal bundle, *SLFt* Superior longitudinal fasciculus temporal bundle, *UF* Uncinate fasciculus

## Discussion

The current study investigated white matter tracts associated with reading and attentional/executive functioning between three groups: DD + ADHD, DD, and TD. As expected, measures of reading and attentional control were significantly different between groups, with TD showing the highest scores on all measures, while DD and DD + ADHD showed no difference in reading but differed significantly in attentional control (Tables 1 and 2). Mean FA in bilateral temporal and parietal portions of the SLF differed between the DD + ADHD and TD groups, with TD showing the highest mean FA (Tables 3 and 4). However, there were no significant differences in FA between DD and DD + ADHD, nor between DD and TD, within the bilateral SLF, ILF, UF, or CC. Although previous literature has shown positive associations between ADHD and MD in the left SLF, left ILF, and bilateral UF [61, 68, 79], no significant group differences were observed in any tract of interest for mean MD.

As expected, our results support the TD group as superior readers with the strongest attentional control and highest white matter diffusivity bilaterally in the parietal and temporal regions of the SLF [6, 58]. The DD + ADHD group on the other hand displayed the lowest scores on behavioral measures of attentional control and the lowest FA results in bilateral parietal and temporal regions of the SLF. The DD group’s results fell between the DD + ADHD and TD groups on attentional control as well as FA bilaterally in both regions of the SLF, while at the same time showing more similar reading deficits to the DD + ADHD group. Although there were significant differences in reading and attentional control between diagnostic groups, only trends for brain-behavior correlations were observed once corrected for multiple comparisons. TOWRE-CST was signficantly different between groups and there were trends for postive relations between TOWRE-CST and mean FA in the left temporal and parietal regions of the SLF across groups. Furthermore, there was a trend for negative relations between GEC-T, with a higher score indicating worse exectuive funtion, and mean FA in the left temporal and parietal regions and the right parietal regions of the SLF across groups. Taken together, our results begin to suggest that as mean FA in the SLF decreases, so does performance on reading and attentional control measures, proposing a continuous effect of the underlying SLF white matter diffusivity on behavior. It is important to note that this possible continuum effect is only reflected in the effects on brain structure, not on behavioral outcomes as our data did not show significant differences in reading between the DD and DD + ADHD groups.

Accordingly, previous research also has suggested that children with comorbid DD and ADHD may exist as a third phenotype independent from sole DD or ADHD conditions [13–16]. Results from the current study support the suggestion of a dual or specific role of the SLF as potentially underlying such comorbid DD and ADHD symptomatology, whether considered as a dual diagnosis or an independent phenotype. In regards to the tracts of interest investigated within this study, only the SLF has been identified as a primary white matter tract associated with both the dorsal phonological system of the reading network [6] and attentional control network [43]; therefore, the main underlying factor within the comorbid group may be due to top-down and bottom-up effects of attentional control interacting with the external learning environment at hand. Indeed, distractors in high-difficult tasks have shown to be positively associated with reliance on bottom-up processing, whereas distractors in low-difficult tasks have been positively associated with reliance on top-down processing for individuals with ADHD, indicating a detrimental association between distractibility and task difficulty [96]. Moreover, reading ability may rely on the balanced integration of top-down and bottom-up processing. In comparison to typical readers, individuals with DD showed reduced functional connectivity between the neural substrates of top-down and bottom-up processing [97] and reduced activation of frontal and parietal cortical areas associated with the attentional control network during reading tasks [98]. Likewise, individuals with comorbid reading disability and ADHD have shown specific deficits in frontal regions within the frontal-striatal pathway [48], which has been functionally related to impairments in executive functioning [47, 49], an ability central to the attentional control network. Nonetheless, Langer and colleagues [49] have further associated specific grey matter deficits within the reading network to a reduced reading ability in comorbid reading disability and ADHD individuals.

Given the profound reading impairment within individuals with DD, the highly demanding task of reading may be further impeded by a dysfunctional attentional control network that moderates top-down and bottom-up processing. This suggests that the SLF, critically employed within both the reading and attentional control networks, may be a main contributor for the successful integration of top-down and bottom-up processing necessary for efficient reading and attentional control. Therefore, one of the primary deficits associated with the comorbid DD and ADHD (particular to ADHD-I) phenotype may be attributed to this additive effect of reduced diffusivity of the SLF, constituting the continuum of FA found within our sample. The consequences of differential white matter integrity in the SLF may be a fundamental factor in attentional control, with DD + ADHD exhibiting poor attentional control and TD exhibiting superior attentional control. Accordingly, a conjunction analysis of studies assessing grey matter differences within ADHD and DD populations found reduced grey matter volume only within the right caudate nucleus of the striatum [50], which compliments the sole finding of reduced grey matter volume within regions of the frontal-striatal pathway for individuals with comorbid reading disability and ADHD [48]. However, as seen in the behavioral measures of reading, the FA differences found within the SLF may not be explicitly additive to reading impairments in DD + ADHD individuals, as the reading network is comprised of several white matter tracts outside the attentional control network [2].

Similarly, white matter diffusivity of the ILF has been associated with both attentional control and reading. Encompassing language systems of logographic phonological alphabets, adults with ADHD [68] and children with DD [59] have been shown to have decreased FA in the ILF compared to controls. In our sample, there was a trend for FA group differences within the ILF similar to the continuum effect shown in the SLF. This may support previous evidence of the effect of ADHD on white matter diffusivity beyond those tracts directly associated with the attentional control network [68]. Although this effect may be detrimental in the case of top-down and bottom-up processing attributed to the attentional control network for individuals with DD and/or ADHD, the suggested third phenotype of comorbid DD + ADHD may show different properties of white matter tract diffusivity within the attentional control network, yet similar diffusivity within the reading network.

Contrary to previous findings concerning white matter diffusivity for individuals with DD [59, 61, 68, 72, 73, 75, 78, 79], mean FA for the ILF, UF, and CC were not significantly different between the DD and TD groups, nor between the DD + ADHD and TD groups. Accordingly, previous research has shown DD to be associated with a *decrease* in FA within the UF [72], whereas ADHD research has shown an *increase* in FA within the same tract [61, 73]; however, there were no statistically significant differences between the DD and DD + ADHD groups in the UF in the current study. If there is a continuum effect in the UF that is similar to that observed in the SLF, with the lowest performing readers having decreased white matter diffusivity, this impact may be counteracted in the current sample due to the potential inverse effects of comorbid ADHD on diffusivity properties. Therefore, shared genetic influences between ADHD and DD [14, 15] may impose potential differing effects on white matter within the UF and ILF (i.e., the reading network) yet potential additive effects within the SLF (i.e., the attentional control network) that exist on a continuum, with DD lying in between DD + ADHD and TD.

### Limitations

Although decreased white matter diffusivity in the CC has been considered an integral component underlying the DD phenotype [75, 76] and has also been observed in individuals with ADHD [78, 79], no significant results indicating group differences for FA were found. Outside the previously discussed additive and counteracting effects of DD and ADHD on white matter, these results may be mainly due to the tract segmentation methods used in the current study. TRACULA utilizes a broad extraction of whole tract mean FA, yet evidence suggests that differences in white matter diffusivity for individuals with DD may lie in smaller regions of the CC [75]. Following the segmentation of the CC into smaller regions, previous research has shown that individuals with DD have increased mean FA in the splenium [99, 100] and have an abnormally shaped splenium, rostrum, genu, and body of the CC [101]. Therefore, a limitation of the current study may be the use of TRACULA, as it utilizes probabilistic tractography for segmenting tracts of interest and for quantifying white matter diffusivity. It is possible that group differences between our tracts of interest may only be found in smaller segments within the tract; therefore, performing a region of interest analysis may provide different results in tracts such as the ILF, UF, and CC. However, TRACULA utilizes individualized subject-specific anatomical landmarks, which allows for the reliable reconstruction of white matter pathways without manual intervention that potentially decreases researcher bias [102]. This is particularly important in the current sample, as other atlas-based tractography methods use adult standardized templates that may not be appropriate for the developing brain.

Similarly, there were no significant differences observed in MD between groups for any of the tracts of interest. Although changes in MD have been reported in individuals with ADHD, there is less evidence of difference in MD associated with DD. Differences between ADHD and typically developing controls is most frequently reported in adult ADHD populations [61, 68]. These differences may be in part to the limited age range (i.e., 7 to 11 years) represented in the current sample. Although most rapid microstructural changes occur in the first 24 months of age, maturation rates in diffusivity measures have been shown to progress at different rates, with MD changes developing much slower than FA [103–105]. This is particularly true for tracts associated with language and cognitive processing such as the SLF and UF. Differences in FA but not MD observed in the current study may have been, in part, due to maturational differences in the diffusion measures used.

Finally, the absence of an ADHD-only comparison group is a potential limitation of the current study. However, our primary aim was to investigate the differences in DD populations with and without ADHD to better understand the differences and similarities of the co-morbid condition in comparison to the’pure’ DD. Due to the nature of the overarching study, we were unable to collect a pure ADHD sample, which prevented us from directly assessing the impact of attentional deficits alone on white matter diffusivity. Future research will benefit from the inclusion of all four potential groups: TD, DD only, ADHD only, and DD + ADHD co-morbid samples. Similarly, future research should further evaluate the contribution of comorbidity to DD observed in ideographic language.

## Conclusions

Our results indicate that neurostructural differences in the SLF may occur in children with DD + ADHD in comparison to TD. Results suggest that differences in white matter diffusivity may exist on a continuum, with DD + ADHD having the lowest mean FA compared to DD only or TD groups. Differences in FA in these specific tracts may underlie the severity of specific behavioral impairments seen in comorbid DD + ADHD when compared to those children with only DD exclusively.

## Data Availability

The datasets used and/or analyzed during the current study are available from the corresponding author upon reasonable request.

## Abbreviations

ADHD: Attentional deficit/hyperactivity disorder
ADHD-C: Comorbid inattentive hyperactive subtype of attentional deficit/hyperactivity disorder
ADHD-I: Inattentive subtype of attentional deficit/hyperactivity disorder
BRI-T: Behavioral Regulation Index T Score taken from BRIEF
BRIEF: Behavior Rating Inventory of Executive Function
CC: Corpus callosum
DBRS: Disruptive Behavior Rating Scale
DD: Developmental dyslexia
DD + ADHD: Developmental dyslexia comorbid with attention deficit/hyperactivity disorder
DTI: Diffusion tensor imaging
FA: Fractional anisotropy
Fmajor: Posterior CC; forceps major
Fminor: anterior CC; forceps minor
GEC-T: Global Executive Composite T Score taken from BRIEF
IFG: Inferior frontal gyrus
ILF: Inferior longitudinal fasciculus
IPL: Inferior parietal lobule
L: Left
MFG: Middle frontal gyrus
R: Right
SLF: Superior longitudinal fasciculus
SLFp: Superior longitudinal fasciculus parietal bundle
SLFt: Superior longitudinal fasciculus temporal bundle
SWAN: Strengths and Weaknesses of ADHD symptoms and Normal Behavior
TD: Typical developing, unimpaired readers
TOWRE-CST: Test of Word Reading Efficiency 2nd Edition Composite Score
TPA: Temporal parietal area
UF: Uncinate fasciculus
WASI-II: Wechsler Abbreviated Scale of Intelligence II
WJ3-Basic: Woodcock Johnson-III Basic Reading Composite Score
